# Supplementary material for: Effects of Sarcosine (N-methylglycine) on NMDA (N-methyl-D-aspartate) Receptor Hypofunction Induced by MK801: In Vivo Calcium Imaging in the CA1 Region of the Dorsal Hippocampus
Source: Brain Sci. 2024 Nov 16;14(11):1150. doi: 10.3390/brainsci14111150 (PMC11592077; doi:10.3390/brainsci14111150)
Supplement: Supplementary file 1 [file brainsci-14-01150-s001.zip › Supplementary Figure S2.pdf]

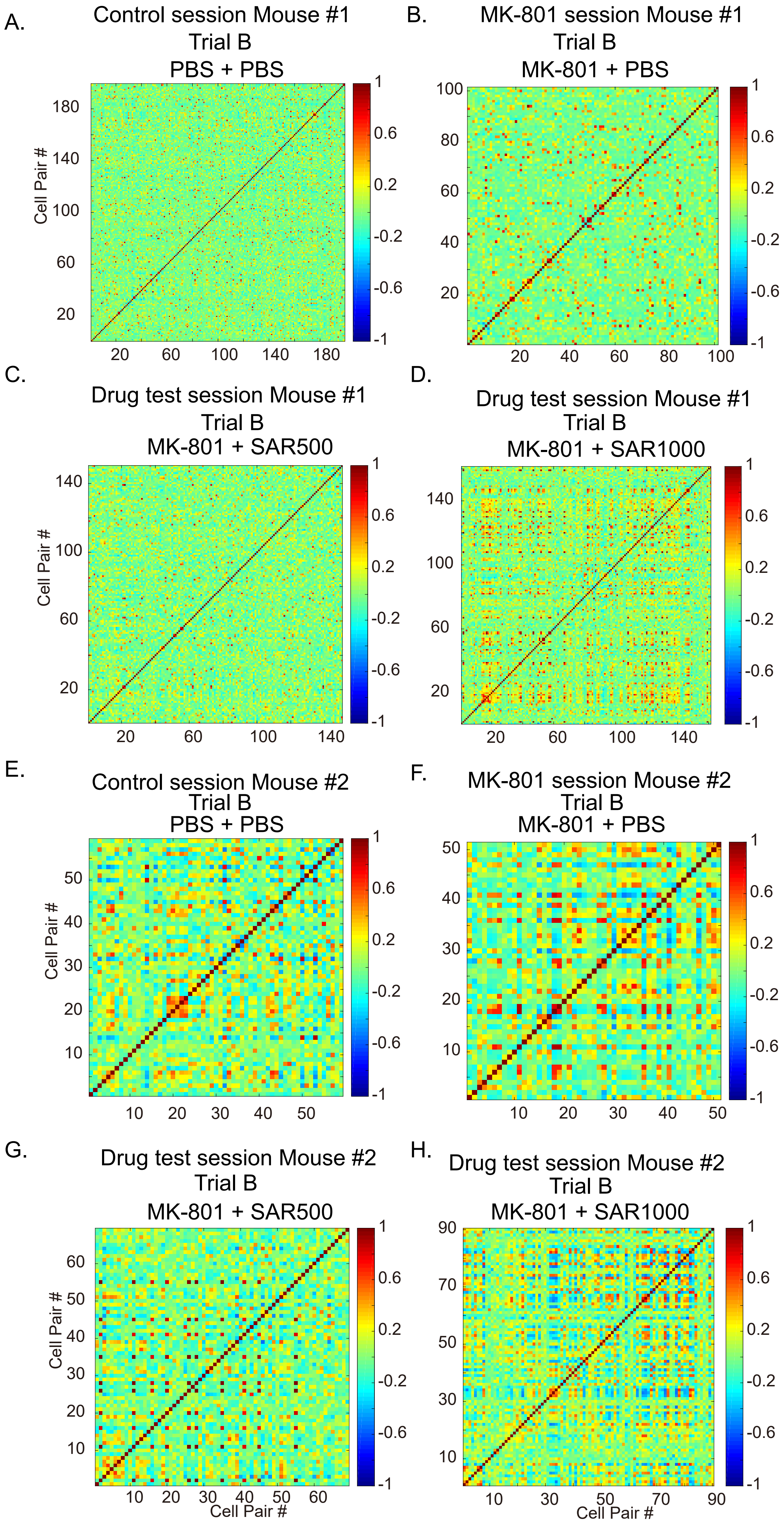
A.
Control session Mouse #1
Trial B
PBS + PBS
B.
MK-801 session Mouse #1
Trial B
MK-801 + PBS
C.
Drug test session Mouse #1
Trial B
MK-801 + SAR500
D.
Drug test session Mouse #1
Trial B
MK-801 + SAR1000
E.
Control session Mouse #2
Trial B
PBS + PBS
F.
MK-801 session Mouse #2
Trial B
MK-801 + PBS
G.
Drug test session Mouse #2
Trial B
MK-801 + SAR500
H.
Drug test session Mouse #2
Trial B
MK-801 + SAR1000
Cell Pair #
Cell Pair #
