# Supplementary figures and images for: Effects of Sarcosine (N-methylglycine) on NMDA (N-methyl-D-aspartate) Receptor Hypofunction Induced by MK801: In Vivo Calcium Imaging in the CA1 Region of the Dorsal Hippocampus
Source: Brain Sci. 2024 Nov 16;14(11):1150. doi: 10.3390/brainsci14111150 (PMC11592077; doi:10.3390/brainsci14111150)

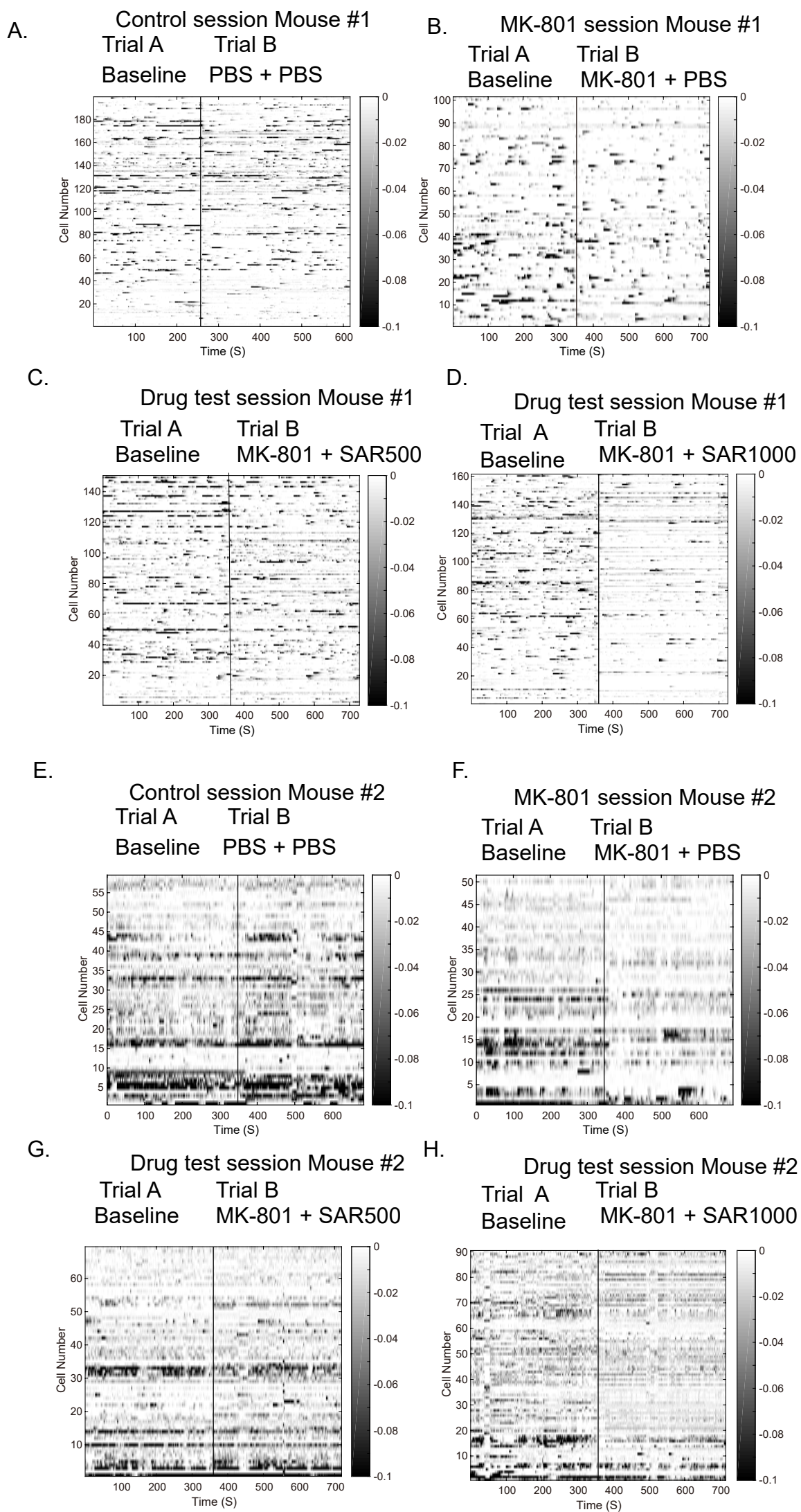

Supplement: Supplementary file 1 [file brainsci-14-01150-s001.zip › Supplementary Figure S1.pdf]
